# Transcriptional Regulation and Protein Localization of Zip10, Zip13 and Zip14 Transporters of Freshwater Teleost Yellow Catfish *Pelteobagrus fulvidraco* Following Zn Exposure in a Heterologous HEK293T Model

**DOI:** 10.3390/ijms23148034

**Published:** 2022-07-21

**Authors:** Sheng-Zan Liu, Yi-Chuang Xu, Xiao-Ying Tan, Tao Zhao, Dian-Guang Zhang, Hong Yang, Zhi Luo

**Affiliations:** 1 Hubei Hongshan Laboratory, Fishery College, Huazhong Agricultural University, Wuhan 430070, China; lsz2020@webmail.hzau.edu.cn (S.-Z.L.); xuyichuang@webmail.hzau.edu.cn (Y.-C.X.); txy7933@mail.hzau.edu.cn (X.-Y.T.); zhaotao2017@webmail.hzau.edu.cn (T.Z.); zdg@webmail.hzau.edu.cn (D.-G.Z.); yang-hong@webmail.hzau.edu.cn (H.Y.); 2 Laboratory for Marine Fisheries Science and Food Production Processes, Qingdao National Laboratory for Marine Science and Technology, Qingdao 266237, China

**Keywords:** micronutrients, zinc homeostasis, SLC39A transporter, transcriptional regulation, subcellular localization, vertebrates

## Abstract

Zip family proteins are involved in the control of zinc (Zn) ion homeostasis. The present study cloned the promoters and investigated the transcription responses and protein subcellular localizations of three LIV-1 subfamily members (*zip10*, *zip13*, and *zip14*) from common freshwater teleost yellow catfish, *Pelteobagrus fulvidraco,* using in vitro cultured HEK293T model cells. The 2278 bp, 1917 bp, and 1989 bp sequences of *zip10*, *zip13*, and *zip14* promoters, respectively, were subcloned into pGL3-Basic plasmid for promoter activity analysis. The pcDNA3.1 plasmid coding EGFP tagged pfZip10, pfZip13, and pfZip14 were generated for subsequent confocal microscope analysis. Several potential transcription factors’ binding sites were predicted within the promoters. In vitro promoter analysis in the HEK293T cells showed that high Zn administration significantly reduced the transcriptional activities of the *zip10*, *zip13*, and *zip14* promoters. The −2017 bp/−2004 bp MRE in the *zip10* promoter, the −360 bp/−345 bp MRE in the *zip13* promoter, and the −1457 bp/−1442 bp MRE in the *zip14* promoter were functional loci that were involved in the regulation of the three *zips*. The −606 bp/−594 bp KLF4 binding site in the *zip13* promoter was a functional locus responsible for zinc-responsive regulation of *zip13*. The −1383 bp/−1375 bp STAT3 binding site in the *zip14* promoter was a functional locus responsible for zinc-responsive regulation of *zip14*. Moreover, confocal microscope analysis indicated that zinc incubation significantly reduced the fluorescence intensity of pfZip10-EGFP and pfZip14-EGFP but had no significant influence on pfZip13-EGFP fluorescence intensity. Further investigation found that pfZip10 localizes on cell membranes, pfZip14 colocalized with both cell membranes and lysosome, and pfZip13 colocalized with intracellular ER and Golgi. Our research illustrated the transcription regulation of *zip10*, *zip13*, and *zip14* from *P. fulvidraco* under zinc administration, which provided a reference value for the mechanisms involved in Zip-family-mediated control of zinc homeostasis in vertebrates.

## 1. Introduction

Micronutrient zinc (Zn) is an essentially functional and structural element for all living organisms, including fish [1]. Both a dietary deficiency and an excess of this micronutrient are deleterious to the growth and development of fish [2]. Zinc deficiency results in growth repression and zinc excess results in toxicity for many fish [3]. Waterborne zinc could also cause chronic toxicity for rainbow trout and other fish [4]. Thus, intracellular free Zn^2+^ must be maintained at a required reasonable level by various Zn transporters, buffering metallothioneins, and sequestering vesicles [5]. For example, in the zebrafish, Zip6 is required for zinc homeostasis maintenance in T-cell development [6] and pigmented cells store zinc and maintain zinc homeostasis through Znt8 [7]. In rainbow trout, MT and other zinc-binding proteins contribute to normal zinc homeostasis in liver cells [8]. Zn transporters, which are SLC39A (Zip) family members, control Zn influx that is assimilated from the extracellular space or released from the intracellular compartment to the cytoplasm. Among the Zn transporters are three LIV-1 subfamily members (Zip10, Zip13, and Zip14) that are responsible for the increment of Zn^2+^ concentration within the cytoplasm of many vertebrates. Each of the three Zip proteins usually forms a heterodimer with another protein and thus plays a functional role. For example, Zip10 forms a heterodimer with Zip6 to facilitate zinc flux to trigger mitosis [9]. Zip13 possesses some domains that are not found in other LIV-1 family members and Zip13 itself may form a homodimer [10]. Aydemir et al. [11] reported that Zip14 might act as a heterodimer with Zip8 to maintain an optimum zinc concentration in mitochondria. Due to these specialties of Zip10, Zip13, and Zip14, we considered it interesting and valuable to explore their transcription regulation. In some vertebrates, several studies explored the responses of these proteins to Zn. For instance, Croxford et al. [12] reported that the *zip10* mRNA level was not influenced by Zn^2+^ deficiency, but the protein level of Zip10 were downregulated in response to zinc deficiency in mouse testes. Zheng et al. [13] reported that in zebrafish gill, zinc excess reduced the mRNA expression level of *zip10* and that zebrafish Zip10 acts as a zinc importer for *X. laevis* oocytes. Lichten et al. [14] reported that high Zn incubation downregulated the *zip10* mRNA expression level in mouse hepatocytes, the AML12 cell line, and the neuro 2a cell line. Further investigation revealed that the mouse mZip10 localized to the AML12 cell membrane and functioned as a Zn^2+^ importer, and that Zn deficiency enriched its localization in the cell membrane [14]. Lee and Bin [15] reported that hZip13 was located on the Golgi of hMSCs and directly mediated Zn homeostasis. Fukada et al. [16] reported that Zip13 disturbance could result in the disorder of intracellular Zn homeostasis. Jeong et al. [17] reported that Zn deficiency elevated the *zip13* mRNA and protein level in HeLa cells. Xu et al. [18] reported that Zip13 released zinc from the vesicular store to the cytoplasm that is required for the normal function of ER and that 1 mM of Zn could increase the protein level of dZip13. Liuzzi et al. [19] reported that Zip14 was located in the plasma membrane of the hepatocytes and increased the Zn concentration of the hepatocytes in mice with hypozincemia. Kim et al. [20] reported that Zip14 was located in the cell membrane and mediated Zn homeostasis. Recently, in our laboratory, Chen et al. [21] found that high Zn administration reduced the *zip10*, *zip13*, and *zip14* mRNA levels in both the hepatocytes and the intestinal epithelial cells of yellow catfish. Thus, all of these studies indicated that Zip10, Zip13, and Zip14 were regulated by Zn. However, the underlying regulatory mechanisms have received very little attention and remain confusing.

In the eukaryotes, transcription factors initiate and regulate gene expression via their binding locus in the promoter. For example, the TFs of NRF1 and the Smad3 binding site were found in the promoter region of the *troc1* gene and play repressing roles in *troc1* expression [22]. Liang et al. [23] reported that TFs of ACSL1 and ASCL2 transcriptionally regulated the *fam13a* gene through the binding region in its promoter. MTF-1, which possesses six Cys2-His2 zinc finger structures specializing in the binding of Zn, is a crucial transcription factor for inducing the transcription of some genes that are associated with Zn homeostasis [24]. MTF-1 first binds with Zn in the cytoplasm and then translocates into the nucleus, where it binds to a specific sequence, MRE, and regulates its target genes [25,26]. MRE elements are present in the promotor regions of many Zn transporters, such as the *znt1*, *znt2*, *znt6*, *znt8*, *zip3*, and *zip8* transporters of *P. fulvidraco* [27,28], the *zip10* transporter of zebrafish [14], and the *znt1*, *znt2*, and the *znt5* transporter in mammals [29,30,31]. Krüppel-like factor 4 (KLF4) is another crucial zinc finger transcription factor [32]. Liuzzi et al. [33] found that Zn deprivation increased the binding of KLF4 to the *zip4* promoter of mice and, thus, regulated the adaptive expression of *zip4*. Other studies concerning KLF4 and *zip13* led us to speculate that there is an enigmatic connection between KLF4 and Zn homeostasis that is mediated by *zip13* [16,34]. The signal transducer and activator of transcription 3 (STAT3) belongs to the JAK/STAT signaling pathway family. STAT3 possesses DNA-binding activity and is capable of interacting with enhancer elements in the promoters of downstream genes [35]. Studies suggested that Zn increased the gene expression level of STAT3 [36] but suppressed the STAT3 signaling cascade [37]. Cousins et al. [38] reported that phosphorylated STAT are capable of regulating the transcription of *zip14*. Thus, it is valuable to investigate whether these transcription factors mediate the transcriptional regulation of *zip* members *zip10*, *zip13*, and *zip14*.

Compared with terrestrial animals that sequester Zn from their diets, aquatic fish possess two pathways for obtaining Zn from diets or from a water environment [39]. Thus, it is worth exploring the regulatory mechanisms in fish through which Zn homeostasis is regulated. Yellow catfish, *Pelteobagrus fulvidraco*, are found broadly across Asian countries and provide an excellent model for nutrition research, due to their delicious flavor, high nutritional value, and economic value [40,41]. Recently, we characterized the full-length cDNA sequences of *zip10*, *zip13*, and *zip14* of yellow catfish [21]. The current study investigates their transcriptional regulation, their responsive mechanisms, and their protein subcellular localization in response to Zn. We used HEK293T as our host cells, because they are reliable model cells for investigating the regulatory mechanisms of the promoters [41], the gene 3′-UTR [42], ER stress [43], and the lipid metabolism [44] of *P. fulvidraco*. Our study illustrated the zinc-responsive transcription regulation mechanisms of *zip10*, *zip13*, and *zip14* and provides some views regarding Zip-family-mediated zinc homeostatic control in vertebrates.

## 2. Results

### 2.1. Cloning and Sequence Analysis of zip10, zip13, and zip14 Promoters of Yellow Catfish

**Figure 1:**
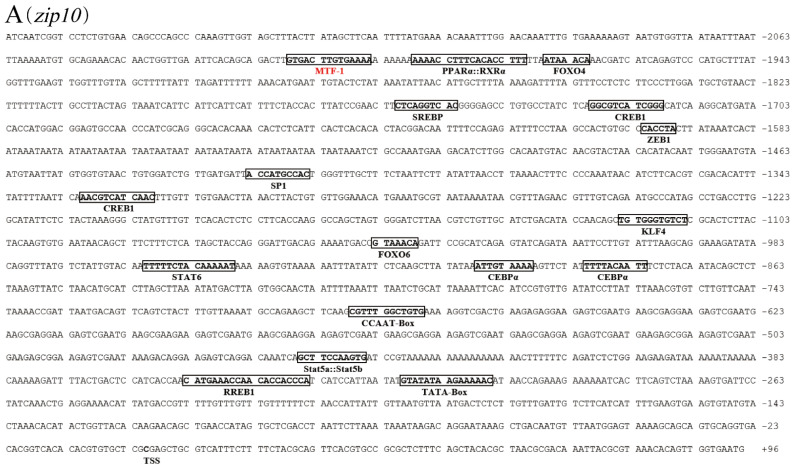

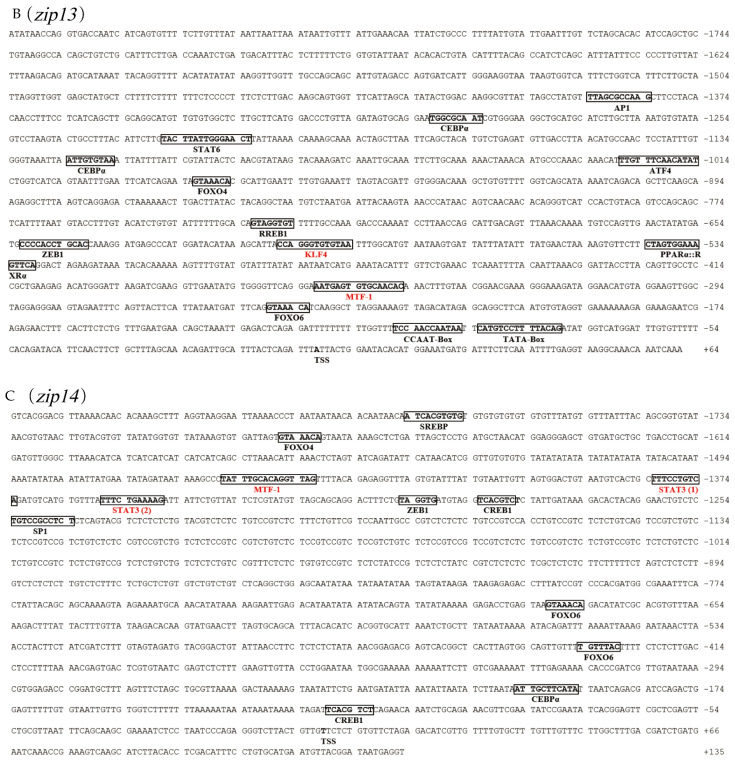
The nucleotide sequences of the *zip10*, *zip13*, and *zip14* promoters of *P. fulvidraco*. (**A**) *zip10*; (**B**) *zip13*; (**C**) *zip14*. Numbers represent the relative distance from the corresponding position to the transcription start site (TSS). The putative TFBSs are boxed in the rectangle. The sequences above the red characters represent the MRE, the KLF4 binding site, and the STAT3 binding site of these promoters.

The 2278 bp, 1917 bp, and 1989 bp sequences of yellow catfish *zip10*, *zip13* and *zip14* promoters, respectively, were cloned successfully, and several putative TFBSs were predicted, including the binding sites of MRE, PPARα:RXR, FOXO4, FOXO6, SREBP, CREB1, AP1, ZEB1, RREB1, SP1, KLF4, STAT3, STAT5a:STAT5b, STAT6, CEBPα, ATF4, CCAAT-box, and TATA-box. The relative positions of the predicted putative TFBSs are listed in Table 1. The nucleotide sequence of the *zip10*, *zip13*, and *zip14* promoters are presented in Figure 1.

### 2.2. 5′-Sequence Deletion Mutation Assays of zip10, zip13, and zip14 Promoters of Yellow Catfish

**Figure 2:**
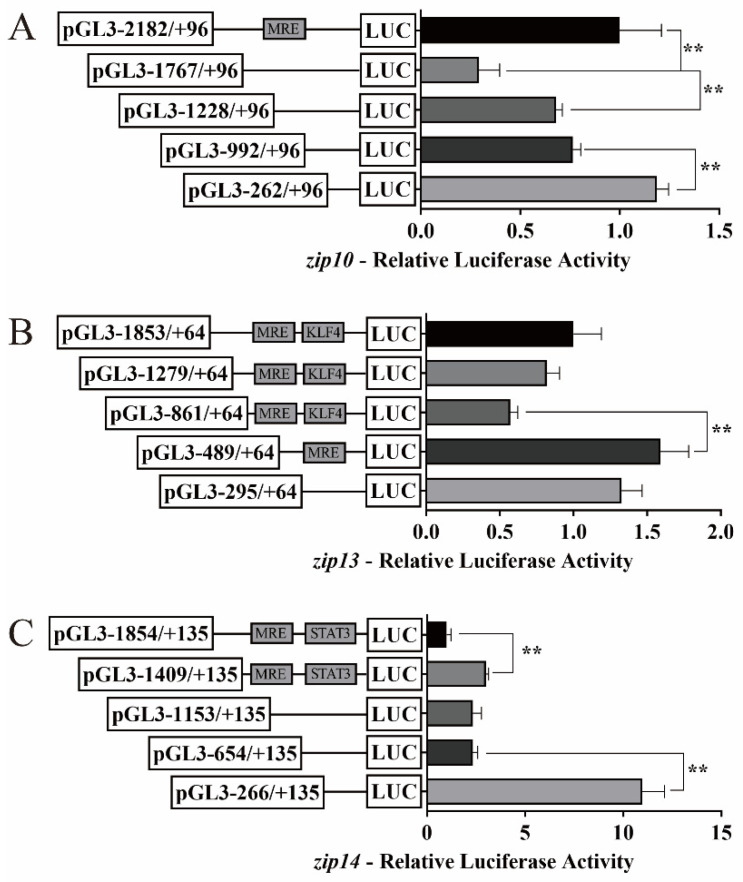
5′-unidirectional deletion assays of *zip10*, *zip13*, and *zip14* promoters of *P. fulvidraco*. (**A**) *zip10*; (**B**) *zip13*; (**C**) *zip14*. Values mean the ratio of Firefly to Renilla luciferase activities, normalized to the control plasmid. The putative MRE, KLF4 binding site, and STAT3 binding site boxed in the rectangle indicate that they were not deleted by mutation. Results are presented as mean ± SEM (*n* = 3). Student *t*-test was used to evaluate the difference. Asterisks (**) mean significant differences between the two groups (*p* < 0.05).

We investigated the activities of the *zip10*, *zip13*, and *zip14* promoters using in vitro cultured HEK293T cells and characterized their functional regions (Figure 2). The deletion of the sequence from −2182 bp to −1767 bp significantly decreased the luciferase activity of the *zip10* promoter, while the deletion of the sequence from −1767 bp to −1228 bp and from −992 bp to −262 bp increased that activity (Figure 2A). The deletion of the sequence from −861 bp to −489 bp significantly enhanced the luciferase activity of the *zip13* promoter (Figure 2B). The sequence deletion from −1854 bp to −1409 bp and from −654 bp to −266 bp increased the luciferase activity of the *zip14* promoter (Figure 2C).

**Figure 3:**
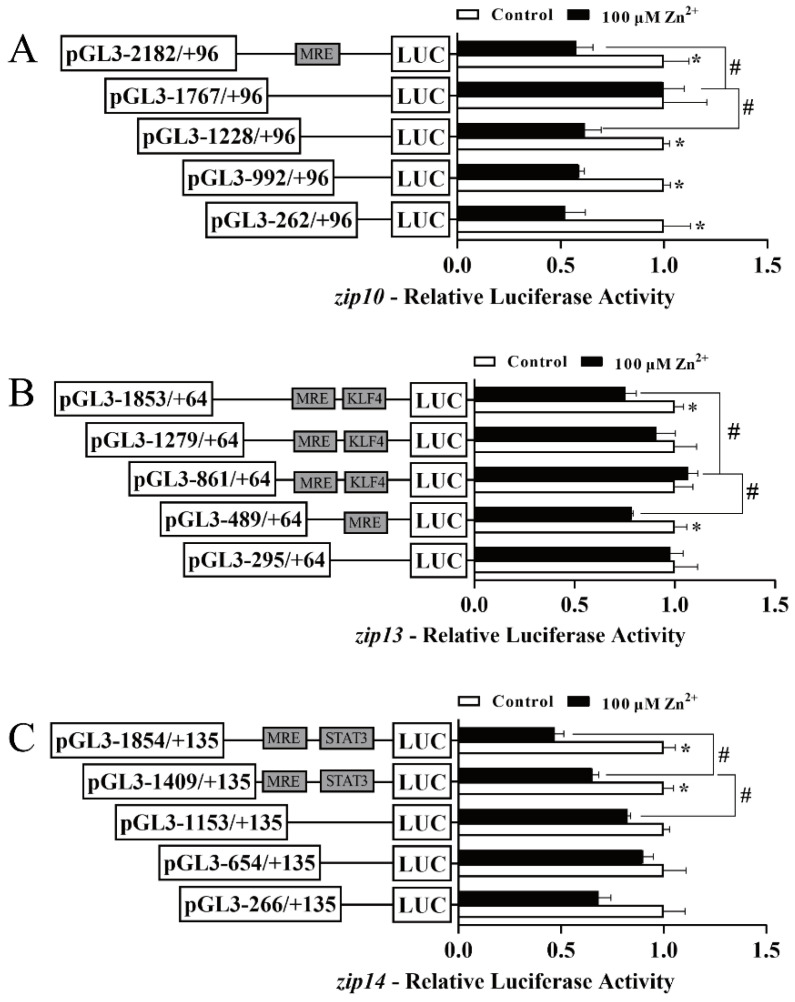
5′-unidirectional deletion assays of the *zip10*, *zip13*, and *zip14* promoters of *P. fulvidraco* after Zn^2+^ administration. (**A**) *zip10*; (**B**) *zip13*; (**C**) *zip14*. Values show the ratio of Firefly to Renilla luciferase activities, normalized to the control. The putative MRE, the KLF4 binding site, and the STAT3 binding site boxed in the rectangle indicate that they were not deleted by mutation. Results are presented as mean ± SEM (*n* = 3). Student *t*-test was used to evaluate the difference. Asterisk (*) indicates significant differences between the control and the Zn-incubated group (*p* < 0.05); hashtag (#) indicates significant differences between two 5′-unidirectional deletion plasmids under the same treatment (*p* < 0.05).

Next, we explored the responses of the *zip10*, *zip13*, and *zip14* promoters to Zn^2+^ administration (Figure 3). For the *zip10* promoter, compared with the control, Zn incubation significantly decreased the luciferase activity from −2182 bp to +96 bp and from −1228 bp and +96 bp. However, compared with the wildtype plasmid, the sequence deletion from −2182 bp to −1767 bp significantly alleviated the Zn-responsive regulatory effect and the sequence deletion from −1767 bp to −1228 bp significantly abolished that effect. These results indicated that Zn has a negative regulatory effect on the *zip10* promoter (Figure 3A). For the *zip13* promoter, compared with the control, Zn incubation markedly decreased the luciferase activity from −1853 bp to +64 bp and from −489 bp to +64 bp. Compared with the wild-type plasmid, Zn incubation markedly alleviated the Zn-induced regulatory effect from −1853 bp to −861 bp. These results suggested that Zn negatively regulates the two areas on the *zip13* promoter (Figure 3B). For the *zip14* promoter, compared with the control, Zn significantly decreased the relative luciferase activity of −1854 bp to +135 bp and −1409 bp to +135 bp. Compared with wild type plasmid, Zn incubation significantly alleviated the negative regulatory effect from −1854 bp to −1409 bp and from −1409 bp to −1153 bp. These results suggest that the two areas on the *zip14* promoter negatively responded to Zn (Figure 3C).

### 2.3. Site-Mutation Assays of the MTF-1, KLF4, and STAT3 Binding Sites in the zip10, zip13 and zip14 Promoters of Yellow Catfish

**Figure 4:**
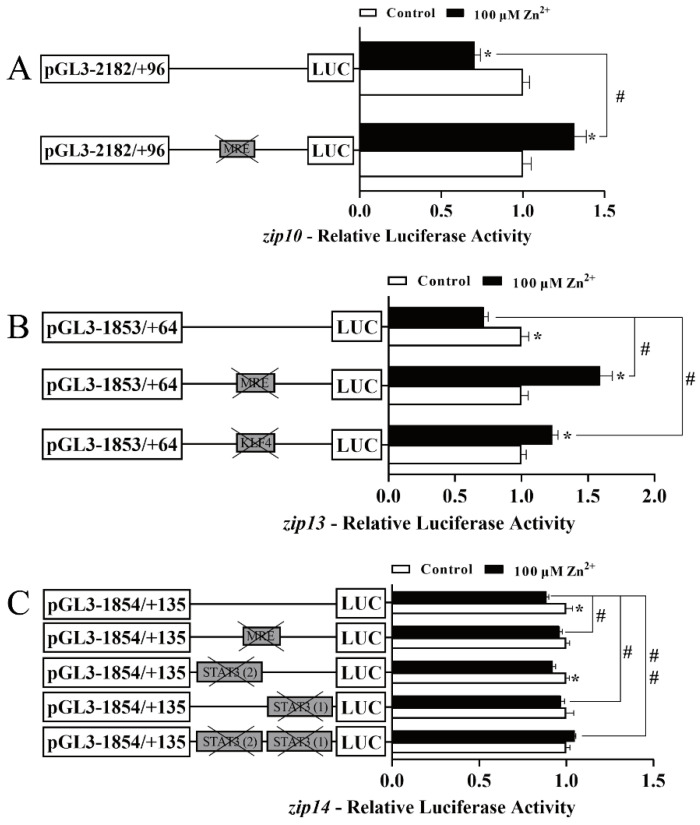
Assays of the predicted MTF-1, KLF4, and STAT3 binding sites in the *zip10*, *zip13*, and *zip14* promoters of *P. fulvidraco* after site-directed mutagenesis. (**A**) Site mutagenesis of MTF-1 on the pGl3-*zip10*-2078 vector; (**B**) site mutagenesis of MTF-1 and KLF4 on the pGl3-*zip13*-1917 vector; (**C**) site mutagenesis of MTF-1 and STAT3 on the pGl3-*zip14*-1989 vector. Values mean the ratio of activities of Firefly to Renilla luciferase, normalized to the control. Results are presented as mean ± SEM (*n* = 3). Student *t*-test was used to evaluate the difference. Asterisk (*) indicates significant differences between the control and the Zn group (*p* < 0.05); hashtag (#) indicates significant differences between two 5′-unidirectional deletion plasmids under the same treatment (*p* < 0.05).

We carried out site-mutation analysis to determine the functional TFBS in the *zip10*, *zip13*, and *zip14* promoters of yellow catfish using in vitro cultured HEK293T cells (Figure 4). The sequences of the selected predicted TFBSs are shown in Figure S1. The bigger a deoxynucleotide is, the more conservative the deoxynucleotide is among vertebrates. For the *zip10* promoter, compared with the wild-type plasmid, the mutation of the putative MRE site (−2004 bp/−2017 bp) markedly increased the luciferase activity of the *zip10* promoter after 100 µM Zn^2+^ incubation (Figure 4A), suggesting the negative regulatory effect by this MRE site in response to high Zn^2+^ administration. For the *zip13* promoter, compared with the wild-type plasmid, the mutation of the putative MRE site (−345 bp/−360 bp) or the KLF4 binding site (−640 bp/−651 bp) significantly increased the luciferase activity of the *zip13* promoter after 100µM Zn^2+^ incubation (Figure 4B), indicating that the MRE and KLF4 binding sites negatively regulated the transcriptional activity of the *zip13* promoter in response to high Zn^2+^ administration. For the *zip14* promoter, compared with the wild-type plasmid, the mutation of the putative MRE (−1442 bp/−1457 bp) site or the mutation of the STAT3 (1) binding site (−1383 bp/−1375 bp) markedly increased the luciferase activity of the *zip14* promoter after 100 µM Zn^2+^ incubation. However, the mutation of the STAT3 (2) binding site (−1358 bp/−1348 bp) showed no remarkable effects (Figure 4C). Surprisingly, we found that the decreased percentage of the full-length promoter activity in Figure 4C was lower than that in Figure 3C. Based on the findings of Miller et al. [45], we thought that both full-length promoter activities in Figure 3C and Figure 4C showed a significant decrease under zinc administration, with a confidence level of 95%. Therefore, these results showed that the MRE and the STAT3 (1) binding site can negatively regulate *zip14* promoter activity. However, the STAT3 (2) binding site could not participate in the Zn-responsive *zip14* transcription.

### 2.4. Electrophoretic Mobility Shift Assay

**Figure 5:**
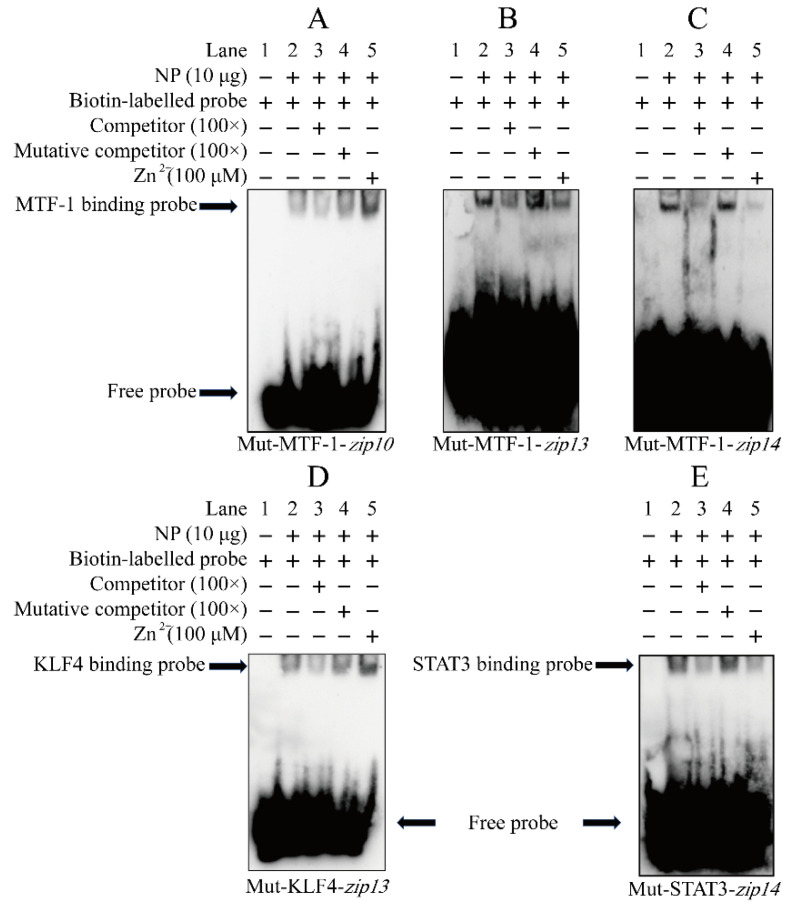
EMSA of the predicted MTF-1, KLF4, and STAT3 binding sequences on the *zip10*, *zip13*, and *zip14* promoters of *P. fulvidraco*. (**A**) MTF-1 binding sites between −2017 bp and −2004 bp in *zip10* promoter; (**B**) MTF-1 binding sites between −360 bp and −345 bp in the *zip13* promoter; (**C**) MTF-1 binding sites between −1457 bp and −1442 bp in the *zip14* promoter; (**D**) KLF4 binding site between −606 bp and −594 bp in the *zip10* promoter; (**E**) STAT3 binding site between −1383 bp and −1375 bp of the *zip14* promoter. NP: nuclear protein. The numbers 1–5 represent the five lanes, respectively.

Next, we carried out EMSA to verify whether these MREs and the binding sites of KLF4 and STAT3 were involved in the regulation on the three *zips* promoters. For the MRE from −2017 bp to −2004 bp on the *zip10* promoter, when the MRE sequences were labeled by the biotin as the probe, we found a bright binding band (lane 2, Figure 5A). Unexpectedly, the 100-fold unlabeled MRE slightly competed for the binding with the NP (lane 3, Figure 5A), and the 100-fold unlabeled unspecific probe alleviated the competition (lane 4, Figure 5A), indicating that −2017 bp/−2004 bp MRE of the *zip10* promoter could bind with the nuclear protein. Zn enhances the brightness of the band (lane 5, Figure 5A), suggesting that this MRE of *zip10* was functional in response to zinc. For the MRE from −345 to −360 bp on the *zip13* promoter, when the MRE sequence was labeled by the biotin as the probe, we found an apparently single binding band (lane 2, Figure 5B). The 100-fold unlabeled MRE probe competed for its binding with the NP (lane 3, Figure 5B), and the 100-fold unlabeled unspecific MRE probe alleviated the competition (lane 4, Figure 5B), implying that −345 bp/−360 bp MRE of the *zip13* promoter could bind with the nuclear protein. However, Zn slightly decreased the brightness of the band, compared with the control (lane 5, Figure 5B). For the MRE from −1457 bp to −1442 bp on the *zip14* promoter, when the MRE sequence was labeled by the biotin as the probe, we found an obvious single binding band (lane 2, Figure 5C). The 100-fold unlabeled MRE probe competed for its binding with the NP (lane 3, Figure 5C), and the 100-fold unlabeled mutated probe alleviated their competition (lane 4, Figure 5C), implying that the −1457 bp/−1442 bp MRE of the *zip14* promoter could bind with the nuclear protein. However, similar to the *zip13* promoter, Zn also decreased the brightness of the binding band, compared with the control (lane 5, Figure 5C). A similar phenomenon was also reported in the promoter region of *mt* [40] and *zip3* [28]. Those results suggested that these putative MRE sites may be involved in the zinc-responsive transcription regulation of the *zip10*, *zip13* and *zip14* promoters For the KLF4 binding sequence from −651 bp to −640 bp on the *zip13* promoter, when the sequence was labeled by the biotin as the probe, we found an apparent binding band (lane 2, Figure 5D). The 100-fold unlabeled sequence competed for the binding with the NP (lane 3, Figure 5D), and the 100-fold unlabeled unspecific probe alleviated their competition (lane 4, Figure 5C), indicating that the −651 bp/−640 bp KLF4 binding site of the *zip13* promoter could bind with the nuclear protein. Meanwhile, Zn also increased the brightness of the band compared with the control (lane 5, Figure 5D), suggesting that the putative KLF4 binding site might participate in the zinc-responsive transcription regulation of the *zip13* promoter. For the STAT3 binding sequence from −1383 bp to −1374 bp on the *zip14* promoter, when the sequence was labeled by the biotin as the probe, we found an apparent binding band (lane 2, Figure 5E). The 100-fold unlabeled MRE competed for the binding with the NP (lane 3, Figure 5E), and the 100-fold unlabeled unspecific probe alleviated their competition (lane 4, Figure 5E), implying that the −1383 bp/−1374 bp STAT3 binding site of the *zip14* promoter could bind with the nuclear protein. Meanwhile, Zn also decreased the brightness of the band, compared with the control (lane5, Figure 5E). The result suggested that the putative STAT3 binding site may participate in zinc-responsive *zip14* transcription regulation. Taken together, our study suggested that these MREs and the binding sites of KLF4 and STAT3 were involved in the regulation on the three *zip* promoters.

### 2.5. Response of pfZip10-EGFP, pfZip13-EGFP, and pfZip14-EGFP to Zn Administration in HEK293T Cells

**Figure 6:**
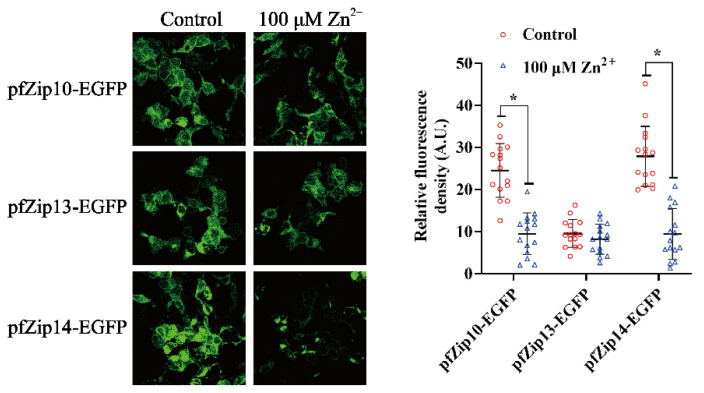
The statistical analysis of the relative fluorescent density of pfZip10-EGFP, pfZip13-EGFP, and pfZip14-EGFP after Zn^2+^ administration. The left panels show the representative image under microscope, with or without Zn incubation. Only one of the fifteen independent experimental results is presented for each group. The right panels show the results, after statistical analysis of the relative fluorescent density. Data were calculated using Image J by setting the upper and lower thresholds. The results were presented as mean ±SEM (*n* = 15) and by scatter diagram. Student *t*-test was used to evaluate the difference. Asterisk (*) indicates significant differences between the control and the Zn group (*p* < 0.05).

Next, we explored whether the pfZip10, pfZip13, and pfZip14 protein expressions were post-translationally regulated by Zn. Due to the absence of available antibodies, we tagged them with the EGFP, which can release the green fluorescence after being excited by ultraviolet light. The analysis method was adopted according to Shihan et al. [46]. In brief, we first used a confocal microscope to catch fluorescent images. Then, the images were inverted into 8-bit one and gray background via Image J. After setting identical upper and lower thresholds for a single EGFP-tagged protein, the images were subjected to calculate the mean fluorescent intensity (Mean). A similar method was also used in other studies [47,48]. The administration of 100 μM Zn significantly reduced the relative green fluorescent intensities of the pfZip10-EGFP and the pfZip14-EGFP, but not that of the pfZip13-EGFP, indicating that the pfZip10 and pfZip14 proteins were post-translationally regulated by Zn (Figure 6).

### 2.6. The Subcellular Localization of the Zip10, Zip13, and Zip14 Proteins of Yellow Catfish in HEK293T Cells

**Figure 7:**
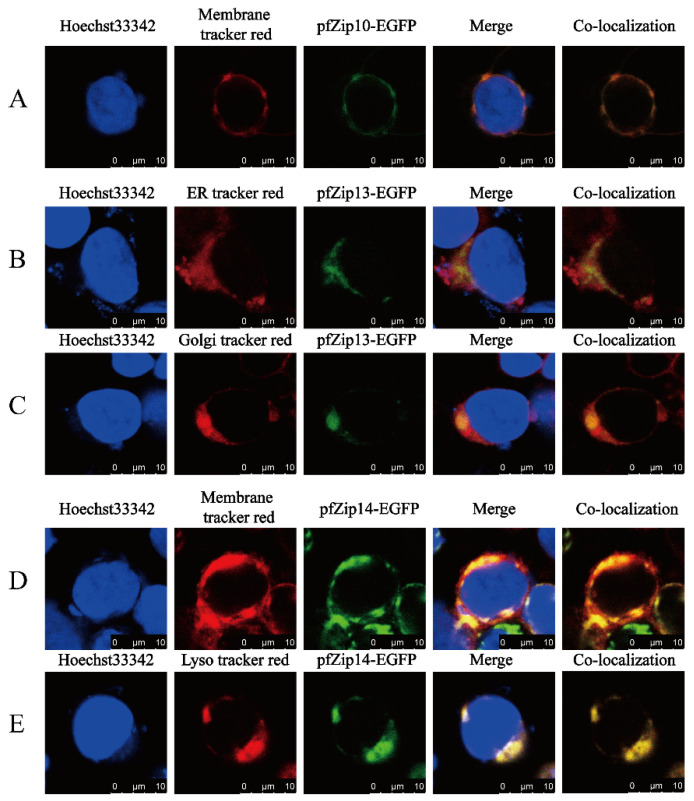
Laser confocal microscope imaging of pfZip10-EGFP, pfZip13-EGFP, and pfZip14-EGFP. Blue, red, and green represents the cell nucleus, the corresponding cell compartment or cytomembrane, and pfZips-EGFP, respectively. The yellow represents the red merged with the green. (**A**) The green overlap with the red indicates the localization of pfZip10 to the cell membrane; (**B**,**C**) The green overlap with the red indicates that pfZip13 colocalizes with the ER and the Golgi; (**D**,**E**) The green overlap with the red indicates that pfZip14 localizes to the cell membrane and colocalizes with the lysosome. Scale labels are 10 μm.

Next, we explored the subcellular localization of pfZip10, pfZip13, and pfZip14 of *P. fulvidraco* by using HEK293T cells. The C-terminal EGFP-tagged pfZip10, pfZip13, and pfZip14 were transfected into human HEK293T cells, and the green fluorescence represented the position of pfZip10, pfZip13, and pfZip14. The membrane tracker (red), the lysosome tracker (red), the ER tracker (red), and the Golgi tracker were applied to mark the positions of the cell membrane, the lysosome, the endoplasmic reticulum, and the Golgi apparatus, respectively. The green fluorescence from the pfZip10-EGFP overlapped with the red fluorescence released by the membrane tracker (red), indicating that pfZip10 was localized to the plasma membrane (Figure 7A). The green fluorescence from the pfZip13-EGFP partially overlapped with the red fluorescence released by both ER tracker (red) and Golgi tracker (red) (Figure 7B,C), indicating that the pfZip13 colocalize with the Golgi apparatus and the endoplasmic reticulum. The green fluorescence released by pfZip14-EGFP also overlapped with the red fluorescence released by the membrane tracker (red) (Figure 7D) and with the red fluorescence released by the lysosome tracker (red) (Figure 7E), indicating that the pfZip14 localize to the plasma membrane and colocalize with the subcellular lysosome.

## 3. Discussion

The promoter region is a short 100–1000 bp DNA fragment upstream from the transcription start site [49]. Chen et al. [40] and Yan et al. [50] reported approximately 2000 bp gene promoters of fish species. Similarly, in our study, 2278 bp, 1917 bp, and 1989 bp *zip10*, *zip13* and *zip 14* promoters of *P. fulvidraco* were cloned. The earlier studies suggested that the core promoters of genes played central roles in initiating the transcriptions [51,52]. In our study, we predicted one GC-box (SP1), one CCAAT-box (NF-Y), and one TATA-box in the core region of the *zip10* promoter. Similarly, Zang et al. [53] reported that SP1 can bind to the human *zip8* promoter and modulate its expression. Langmade et al. [54] reported one SP1 in the mouse *mt1* promoter, and four SP1 in the mouse *znt1* promoter, which are required for their basal expression. Bird et al. [55] reported that the yeast homologous Zn transporter *zrt2* possesses a TATA-box that is required for restraining its expression. The CCAAT-box is a significant component regulated by NF-Y [56]. Our study also indicated that the *zip13* promoter possesses one CCAAT-box and one TATA-box, in agreement with the results reported for the *zip8* and *mt* promoters of *P. fulvidraco* [28,40]. In addition, our study found only one SP1 in the *zip14* promoter. Furthermore, our study predicted some putative TFBSs in their promoters, such as CEBPα, SREBP, PPARα, MTF-1, ATF, KLF, STAT3, RREB1, and ZEB1, which may possess a regulatory function for the three promoters (*zip10*, *zip13*, and *zip14*). These TFBSs have been reported in previously published studies as being within the promoter regions of other genes [27,31,57,58]. In the present study, we chose MTF-1, KLF4, and STAT3 binding sites to explore whether they are functional sites for the regulation of the *zip10*, *zip13*, and *zip14* promoters.

MTF-1 is an intracellular Zn sensor that can bind to the MRE of the promoters of its target genes and activate target genes associated with Zn homeostasis [29]. Recently, Chen et al. [40] found that the MRE existed in the region of the *mt* promoter and was essential for the upregulation of mt expression. Song et al. [41] found that MTF-1 can bind to the MREs of *znt1*, *znt2*, *znt6*. and *znt8* and upregulate their expression. As in our study, the site mutation analysis implied that the mutation of MRE alleviated the decrease in luciferase activity that was caused by high Zn administration. Here, we found that the −2017 bp/−2004 bp MRE in the *zip10* promoter, the −360 bp/−345 bp MRE in the *zip13* promoter, and the −1457 bp /−1442 MRE in the *zip14* promoter were indispensable for the downregulation of *zip10*, *zip13*, and *zip14* expression induced by high Zn administration. Similarly, Chen et al. [21] reported the decreased relative gene expression of the *zip10*, *zip13*, and *zip14* promoters in both hepatocytes and intestinal epithelial cells. Interestingly, in the *zip10* promoter, the MRE deletion showed different activity with the mutated MRE. Deletion mutation deleted a sequence of approximately 400 bp, while site mutation disrupted only the putative binding sequence. Therefore, we speculated that the rest sequence in the deleted 400 bp~ was functionally antagonistic to the MRE in response to zinc. Furthermore, our EMSA results showed that those MREs were capable of binding nuclear protein. High Zn enhanced the binding effect in the *zip10* promoter. However, high-Zn administration showed a relatively low binding affinity in the *zip13* and *zip14* promoters, and a similar phenomenon was observed in the *zip3* and *mt* promoters [28,40]. Lichten et al. [14] showed that knockout of the MTF-1 gene in mice increased the gene expression of the *zip10* promoter, implying that MTF-1 was a functional repressor of the *zip10* promoter under high-Zn treatment. Similarly, Chen et al. [28] found a negative regulatory effect of the MRE on the *zip3* and *zip8* promoters. Taken together, these results indicated the zinc-responsive regulation roles of these MREs in the transcriptional regulation of the *zip10*, *zip13*, and *zip14* promoters.

In our study, we predicted −606 bp/−594 bp KLF4 binding sites within the *zip13* promoter. We found that the mutation of the KLF4 binding site alleviated the Zn-induced decrease in the activity of the *zip13* promoter, suggesting that this KLF4 binding site is necessary for the decrease in *zip13* expression activity in a high-Zn environment. Our EMSA showed that high Zn significantly enhanced the binding effect between nuclear proteins and the KLF4 binding sites. Thus, we confirmed that the KLF4 binding sites could regulate the *zip13* expression in response to a high zinc environment, implying a crucial mechanism whereby KLF4 maintains Zn homeostasis. It was also reported that the KLF4 binding site is responsible for the upregulation of *mtf-1* expression [40]. It seems reasonable to speculate that the KLF4 binding site mediated the MTF-1-induced downregulation of the *zip13* promoter. Liuzzi et al. [33] reported that knockdown of *klf4* restrained the upregulation of the *zip4* promoter in a zinc-depleted environment. Similarly, our results indicated that KLF4 play crucial roles in maintaining intracellular Zn homeostasis in a high-Zn environment through regulating *zip13* expression.

We also predicated the −1383 bp /−1375 bp STAT3 (1) and the −1358 bp /−1348 bp STAT3 (2) binding sites within the *zip14* promoter. The site mutation analysis indicated that STAT3 (1), not STAT3 (2), was responsible for the downregulation of the activity of the *zip14* promoter, and our EMSA confirmed that the effect of the −1383 bp /−1375 bp STAT3 (1) binding site on the *zip14* promoter was the functional one. Thus, our results indicated that STAT3 mediated the maintenance of Zn homeostasis by regulating the *zip14* expression via the −1373/−1383 bp STAT3 (1) binding site on its promoter. Interestingly, Du et al. [59] discovered that STAT3 was activated in response to Zn deprivation and mediated Zn deficiency-induced upregulation of the *zip2* expression. Kitabayashi et al. [37] reported that Zn could attenuate STAT3 activation. Thus, we speculated that Zn alleviated the binding effect of STAT3 to the *zip14* promoter. In addition, other studies suggested that the *zip6* promoter served as the STAT3 target [60,61]. Cousins et al. [38] mentioned that phosphorylated STAT accounted for the expression of the *zip14* promoter Thus, these results, together with ours, suggest that STAT3 plays a crucial role in maintaining Zn homeostasis and that the STAT3 binding site participated in the control of the *zip14* promoter of *P. fulvidraco*.

Within the body, there could be enhancer (or silencer) DNA fragments far away from the transcription start site, and the formation of a chromosome loop makes it possible for the enhancer (or silencer) to participate in enhancing (or repressing) transcription. In our study, we confirmed that the predicted zinc-related TF binding sites were functional and involved in zinc-responsive regulation. Most TF binding sites are a 4 bp- to 30-bp wide integral DNA sequence [62]. Furthermore, the core consensus sequence of the MRE is TGCRCNC, in which R represents A or G and N represents any nucleotide [63]. STAT3-binding sites are characterized by clusters of conserved motifs with a core sequence of TTCT/CNA/GGAA, in which N represents any nucleotide [64]. KLF4 binding sites possess a DNA bind domain of CACCC-box [65]. Therefore, site-mutation was carried out by substituting the predicted conservative sequences with non-conservative ones, according to Jaspar. This may result in low specificity of the experiment. Further study is required to identify the function of a single nucleotide. We highlighted that the promoters are functional in the regulation of the zinc homeostasis of the host HEK293T cells. Referring to our previous study [21], we also speculate that the promoter might participate in the zinc homeostasis control of its native organism, *P. fulvidraco*.

The SLC39A Zn transporters carry Zn into cytoplasm from intracellular organelles or extracellular space, and their specific subcellular localization determines the specific function of the Zn transport. For example, Kong et al. [66] found that maternally derived Zip10 localize to oocytes membrane and help to drive the oocyte-to-egg transition. Jeong et al. [17] reported that the vesicular-localized Zip13 could adjust the dynamic of cytosolic labile Zn levels, which help accumulate Zn into vesicles, causing ER dysfunction and stress. Fukunaka et al. [67] found that the Zip13 located on the Golgi could suppress the biogenesis of beige adipocytes and energy expenditure by regulating the C/EBP-β expression. Kim et al. [20] pointed out that Zn transported by the plasma-membrane-located Zip14 could subdue apoptosis and steatosis by restraining the activity of PTP1B and ER stress. Thus, it is worth exploring the subcellular localization of Zip10, Zip13, and Zip14. In our study, the pfZip10 located on the cell membrane, in agreement with other reports [14,66,68]. Our study indicated that the pfZip13 colocalizes with both the Golgi apparatus and the endoplasmic reticulum. Similarly, Xiao et al. [69] found that the Drosophila dZip13 partially colocalize with the ER/Golgi. In our study, the pfZip14 not only localize to the plasma membrane, but also colocalize with intracellular lysosome. Similarly, Zhao et al. [70] reported that the mouse mZip14 localizes to the basolateral membrane of enterocytes, early endosomes, and lysosomes.

Several studies have established that high Zn reduced the mRNA and protein expression of the *zip10*, *zip13*, and *zip14* promoters [13,14,17,18,21]. Similarly, our fluorescence statistics experiments showed that Zn administration reduced both pfZip10 and pfZip14, but not pfZip13. Lysosome is an organelle involved in the degradation of cell debris and unnecessary protein via endocytosis. Wang et al. [71] reported that Zn repletion could lead exogenously-expressed mouse mZip1-HA and mZip3-HA protein to the endocytosis pathway. Kim et al. [72] reported that mZip4-HA in HEK293 was endocytosed in response to zinc treatment. Their studies suggested that mZip1-HA, mZip3-HA, and mZip4-HA are post-translationally regulated by zinc, which led us to speculate that high zinc could regulate pfZip10, pfZip13, and pfZip14 from the protein level.

## 4. Materials and Methods

### 4.1. Animals, Cells, and Reagents

The tail fins of *P. fulvidraco*, used for genomic DNA extraction, were purchased from a local fishery farm (Wuhan, China). The HEK293T cell lines were from the Cell Resource Center in the Fishery College of Huazhong Agricultural University (HZAU, Wuhan, China). The Dulbecco’s modified eagles medium (DMEM), the 0.25% trypsin-EDTA, and the fetal bovine serum (FBS) were from Gibco (Thermo Fisher Scientific, Waltham, MA, USA). Lipo293 for transfecting plasmid into HEK293T were from Beyotime Biotechnology, Wuhan, China. Passive lysis buffer and the dual luciferase activity assay reagents were from Promega, Madison, WI, USA. Nuclear protein extraction kits were from Thermo Fisher Scientific, Waltham, MA, USA). We made sure that the protocols for animal experiments and the use of cell lines followed the institutional ethical guidelines of HZAU for the care and use of laboratory animals, and that they were approved by the university’s ethics committee (I.D. code: Fish-2020-09-24).

### 4.2. Promoters Cloning and Plasmids Construction

Based on the full-length complementary DNA (cDNA) of the *zip10*, *zip13*, and *zip14* promoters obtained in our laboratory [21], we identified their transcription start sites (TSS) through the RNA ligase-mediated rapid amplification of 5′cDNA ends (RLM-5′ RACE) method that was described in Xu et al. [73]. For constructs for promoters, the specific primers used for nested PCR-generating promoters are listed in Table S1. Then, the *zip10*, *zip13* and *zip14* promoters were subcloned into the pGL3-Basic between the Sac Ⅰ and Hind Ⅲ restriction cloning sites with Clone press II One Step Cloning Kit (Vyzame, Piscataway, NJ, USA), according to the distance from the TSS. Those full-length promoters (wild type) were pGL3-2182/+96 (*zip10*), pGL3-1853/+64 (*zip13*), and pGL3-1854/+135 (*zip14*). The unidirectional deletion mutation products of corresponding promoters were randomly generated using the full-length promoters as templates. The primer sequences are listed in Table S2. According to the distance from the TSS, those deletion mutation promoters were pGL3-1767/+96, pGL3-1228/+96, pGL3-992/+96, and pGL3-262/+96 for *zip10* promoter; pGL3-1279/+64, pGL3-861/+64, pGL3-489/+64, and pGL3-295/+64 for *zip13* promoter; and pGL3-1409/+135, pGL3-1153/+135, pGL3-654/+135, and pGL3-266/+135 for *zip14* promoter. For constructs coding EGFP-tagged pfZips (pfZip10, pfZip13, and pfZip14), the complete cDNA sequences of the *zip10*, *zip13* and *zip14* promoters were generated by the same nested PCR principle. The primers are listed in Table S3. Then, the pfZips’ cDNA end with sequences coding EGFP were subcloned into pcDNA3.1 with the CMV promoter between the Himd III and Xhol restriction cloning sites with a Clone press II One Step Cloning Kit (Vyzame, Piscataway, NJ, USA), and the constructs were pcDNA3.1-pfZip10-EGFP, pcDNA3.1-pfZip13-EGFP, and pcDNA3.1-pfZip14-EGFP.

### 4.3. Promoter Sequence Analysis

For the function characterization of these promoters, we predicted the putative transcription factor bind sites (TFBSs) with the online database MatInspector (http://www.genomatix.de/, accessed on 31 August 2021), with a minimum threshold of 0.85, and Jaspar (http://jaspar.genereg.net/, accessed on 31 August 2021), with minimum threshold of 9. The MegAlign Pro Sequence Alignment Software was used to evaluate whether the sequences were successfully cloned, according to NCBI. (Accession numbers: pfZip10, MK448212.1; pfZip13, MK448215.1; and pfZip14, MK448216.1).

### 4.4. Dual Luciferase Activities Assays

The transfection of the constructed promoter plasmids into the HEK293T cells and the assay of the dual luciferase activities were carried out according to the methods of Xu et al. [73]. Briefly, the HEK293T cells were seeded into 24-well plates and maintained in the DMEM medium with 10% FBS at 5% CO_2_ and 37 °C in a humidified atmosphere until the relative density reached 70% to 80% confluence. Then, the maintaining DMEM was replaced with fresh DMEM (10% FBS) or DMEM (10% FBS) + 100 μM Zn^2+^. The Zn concentration was selected according to our recent study [40]. Four hundred and fifty ng constructed promoter plasmids and 50 ng pRL-TK as control were co-transfected into the HEK293T cells using Lipo 293. Each transfection was conducted in triplicate. Then, after 24 h incubation, the cells were harvested and lysed with the passive lyse buffer for analysis of the dual luciferase activities according to the protocols of the dual-luciferase reporter assay system. We measured the ratio of the Firefly to Renilla luciferase activity.

### 4.5. Site Mutation Analysis of TFBS in the zip10, zip13, and zip14 Promoters

To identify the corresponding binding sites on the *zip10*, *zip13* and *zip14* promoters, we used the QuickChange II Site-Directed Mutagenesis Kit (Vazyme, Piscataway, NJ, USA) to direct the site mutation. The corresponding full-length promoters were used as templates. The specific primers for the site mutation are listed in Table S4, and the plasmids were Mut-MTF-1-*zip10*, Mut-MTF-1-*zip13*, Mut-KLF4-pGL3-*zip14*, Mut-MTF-1-pGL3-*zip13*, and Mut-STAT3-pGL3-*zip14*. Then, the plasmids were transfected and the cells were harvested for dual luciferase activity analysis.

### 4.6. Electrophoretic Mobility-Shift Assay

An EMSA was conducted to verify the MTF-1, KLF4, and STAT3 binding on the *zip10*, *zip13*, and *zip14* promoters. Briefly, the HEK293T cells were seeded into 6-cm dishes and maintained in the DMEM with or without 100 μM Zn^2+^. The incubation lasted for 24 h. Then, the cells were lysed for the extraction of nucleoprotein (NP) and the protein concentration was determined with a bicinchoninic acid assay (BCA) method. Ten μg of nucleoprotein was added and mixed with each oligonucleotide duplex of the binding sequence of the MTF-1, KLF4, and STAT3, using the EMSA kit according to manufacturer’s protocols (Invitrogen, Carlsbad, CA, USA). The oligonucleotide sequences of the EMSA are presented in Table S5.

### 4.7. Fluorescence Intensity Analysis

The transfection protocols of the constructed pcDNA3.1-pfZip10-EGFP, pcDNA3.1-pfZip13-EGFP, and pcDNA3.1-pfZip14-EGFP were the same as that of the promoter plasmids indicated in the Section 2.4; the 500 ng constructed plasmids were transfected. Then, after 24 h, the cells were washed carefully three times with PBS containing 1 mm MgCl2 and 0.1 mm CaCl2. They were fixed with 4% paraformaldehyde for 5 min at room temperature. Next, the cells were again washed carefully three times with PBS. A Leica TCS DMI8 fluorescent microscope was used to catch the green fluorescence images. Image J software was used to quantify the relative fluorescence intensity according to the methods Shihan et al. [46] and Ruggiero et al. [47].

### 4.8. Subcellular Localization Investigation of Zip10, Zip13, and Zip14

We further investigated the pfZips’ subcellular localization. The HEK293T cells were seeded on coverslips, and the transfection of the constructed pcDNA3.1-pfZip10-EGFP, pcDNA3.1-pfZip13-EGFP, and pcDNA3.1-pfZip14-EGFP and the cell-washing were as indicated in the Section 2.4. Then, the membrane tracker (red) (Dil, 1:400), the lysosome tracker (red) (DND-99, 1:1000), the ER tracker (red) (Glipalamides, 1:1000), or the Golgi tracker (red) (C5-ceramide, 1:100) were used to track the cell membrane, the lysosome, the endoplasmic reticulum (ER), or the Golgi apparatus, respectively. To stain the nuclei, the cells were washed with PBS and then incubated in 10 g/mL Hoechst for 10 min. After several washes via PBS, coverslips were mounted on the microscope slides for the confocal imaging, using a Leica TCS SP8 laser-scanning confocal microscope.

### 4.9. Statistical Analysis

We carried out the statistical analysis with SPSS 22.0 software (Armonk, NY, USA). All of the data were presented as means ± standard error of mean (SEM). First, we analyzed the data for normality via the Kolmogorov–Smirnov test, and for the homogeneity of the variances among the treatments via Bartlett’s test. Then, we used one-factor ANOVA and Duncan’s multiple range test to analyze the significance of the differences among ≥3 treatments, and Student’s *t*-test between two treatments; *p* < 0.05 was considered to be statistically significant.

## 5. Conclusions

In summary, for the first time, we characterized the structure and function of the *zip10*, *zip13*, and *zip14* promoters from *P. fulvidraco* in the heterologous expression host HEK293T. We discovered that a high concentration of Zn decreased the activities of the three *zip* promoters. The MRE in the *zip10* promoter, the MRE and KLF4 binding sites in the *zip13* promoter, and the MRE and STAT3 binding sites in the *zip14* promoter mediated the zinc responsive transcription regulation of their activities. We found that the pfZip10 located on the cell membrane, the pfZip13 colocalized with ER and Golgi, and the pfZip14 colocalized with lysosome. A high Zn administration reduced the protein level of pfZip10 and pfZip14. We illustrated the transcription regulation of *zip10*, *zip13*, and *zip14* promoters under zinc administration and provided a novel mechanism for Zip-family-mediated zinc homeostatic control in vertebrates.

## Figures and Tables

**Table 1:** Summary of the relative location of the predicted putative TFBS in the *zip10*, *zip13*, and *zip14* promoters. “None” indicates that there were no predicted corresponding TFBS by setting a threshold of 0.85 in MatInspestor and a threshold of 9 in Jaspar.

Name	Location in *zip10* Genomic Locus	Location in *zip13* Genomic Locus	Location in *zip14* Genomic Locus
MRE	−2017 bp/−2004 bp	−360 bp/−345 bp	−1457 bp/−1442 bp
PPARα:RXR	−1997 bp/−1980 bp	−543 bp/−529 bp	None
FOXO4	−1976 bp/−1970 bp	−981 bp/−975 bp	−1687 bp/−1681 bp
FOXO6	−1043 bp/−1037 bp	−248 bp/−242 bp	−681 bp /−675 bp and −435 bp /−429 bp
SREBP	−1760 bp/−1751 bp	None	−1785 bp/−1776 bp
CREB1	−1729 bp/−1718 bp and −1330 bp/−1319 bp	None	−1293 bp/−1286 bp and −119 bp/−112 bp
AP1	None	−1393 bp/−1383 bp	None
ZEB1	−1601 bp/−1596 bp	−651/−640 bp	−1306 bp /−1301 bp
RREB1	−353 bp/−334 bp	−731 bp/−724 bp	None
SP1	−1423 bp/−1414 bp	None	−1253 bp/−1243 bp
KLF4	−1124 bp/−1114 bp	−606 bp/−594 bp	None
STAT3	None	None	(−1383 bp/−1375 bp and −1358 bp/−1348 bp)
STAT5a:STAT5b	−456 bp/−445 bp	None	None
STAT6	−960 bp/−946 bp	−1126 bp/−1212 bp	None
CEBPα	−890 bp/−881 bp and −907 bp/−899 bp	−1300 bp/−1292 bp and −1123 bp/−1115 bp	−206 bp/−196 bp
ATF4	None	−1027 bp/−1014 bp	None
CCAAT-box	−687 bp/−676 bp	−106/−95 bp	None
TATA-box	−319 bp/−305 bp	−92 bp/−78 bp	None

## Data Availability

The data used to support the findings of this study are available from the corresponding author upon request.

## Supplementary Materials

The following supporting information can be downloaded at: https://www.mdpi.com/article/10.3390/ijms23148034/s1.

## Abbreviations

LZT: LIV-1 subfamily of zinc transporter; MRE: metal responsive element; PPARα: peroxisome proliferator-activated receptor alpha; FOXO: forkhead box protein; SREBP: sterol regulatory element-binding protein; CREB1: cAMP responsive element-binding protein 1; ZEB1: zinc finger E-box-binding homeobox 1; SP1: GC-box; STAT3: signal transducer and activator of transcription 3; KLF4: Krüppel-like factor 4; CEBPα: CCAAT enhancer binding protein alpha; RREB1: ras responsive element-binding protein-1; MTF-1: metal-responsive transcription factor-1; TFBS: transcription factor binding sites; TSS: transcription start sites; DMEM: Dulbecco’s modified eagles medium; FBS: fetal bovine serum; RLM-5′ RACE: RNA ligase-mediated rapid amplification of 5′cDNA ends; EMSA: electrophoretic mobility shift assay; NE: nuclear extract; EGFP, enhanced green fluorescent protein; SEM: standard error of mean; ER: endoplasmic reticulum; Zn: zinc; SLC39A: solute carrier 39A, Zip: protein; *zip*: gene.
