# Supplementary material for: Transcriptional Regulation and Protein Localization of Zip10, Zip13 and Zip14 Transporters of Freshwater Teleost Yellow Catfish Pelteobagrus fulvidraco Following Zn Exposure in a Heterologous HEK293T Model
Source: Int J Mol Sci. 2022 Jul 21;23(14):8034. doi: 10.3390/ijms23148034 (PMC9321221; doi:10.3390/ijms23148034)
Supplement: Supplementary file 1 [file ijms-23-08034-s001.zip › ijms-1733889-supplementary.pdf]

Table S1. Nested PCR primers used for the cloning of *zip10*, *zip13* and *zip14* promoters of *Pelteobagrus fulvidraco*

Genes	Group	Forward primer (5'-3')	Reverse primer (5'-3')
zip10	[1] PCR-1	ACCTTTTAAGTGAAAATTGTGCT	AGTGTGTGAAAACAAAATCGC
	[2] PCR-2	[3]ctatcgataggtaccgagctcATCAATCGGTCCTCTGTGAACAG	cagtaccggaatgccaagcttCATTCACCAACTGTGTTTACGCG
zip13	PCR-1	TTTACACTGGTTAAATATAGGCTT	TTTGATTTGTTTGCCTTACCTCA
	PCR-2	ctatcgataggtaccgagctcATATAACCAGGTGACCAATCATCAGT	cagtaccggaatgccaagcttTTTGATTTGTTTGCCTTACCTCAA
zip14	PCR-1	TTGGAAACTTATAGAATGTGCTT	GCACATCTACAATTCTTTTATCGT
	PCR-2	ctatcgataggtaccgagctcGTCACGGACGTTAAAACAACACA	cagtaccggaatgccaagcttCTTTTATCGTACCTCATTATCCGTAAC

1: The first group of primers used for nested PCR.

2: The second group promers used for nested PCR.

3: The part of “uppercase” are primers to generated promoter sequence, the part of “lowercase” are primers assistting to generate recombination sequence to subcloned to pGL3-basic plasmid.

Table S2. PCR Primers used for 5'-deletion promoter plasmids construction of *zip10*, *zip13* and *zip14* of *Pelteobagrus fulvidraco*

Gene	Primers	Forward primer (5'-3')	Reverse primer (5'-3')
zip10	pGL3－1767/＋96	[5]taccgagctCGAACTTCTCAGGTCACGGGGAGC	cagtaccggaatgccaagcttGGCAAAGTTTCCACACGGAA
	pGL3－1228/＋96	ctatcgataggtaccgagctcGGTTGTGTATAAATGCCAATGCA	cagtaccggaatgccaagcttGGCAAAGTTTCCACACGGAA
	pGL3－992/＋96	ctatcgataggtaccgagctcACACAGCTACTGAATTCCAGTACGTC	cagtaccggaatgccaagcttGGCAAAGTTTCCACACGGAA
	pGL3－262/＋96	ctatcgataggtaccgagctcTGCATGAAGTAACATTTATAGCATTTG	cagtaccggaatgccaagcttGGCAAAGTTTCCACACGGAA
zip13	pGL3－1279/＋64	ctcGCATGCATCTTGCTTAAATGTGTATAGTCC	aagcaagatgcatgcGAGCTCGGTACCTATCGATAGAGAA
	pGL3－861/＋64	ggtaccgagctcACTTATACTACAGGCTAATGTCTAATGAATTAC	ataagtGAGCTCGGTACCTATCGATAGAGAAAT
	pGL3－489/＋64	gataggtaccgagctcTTATATAATAATCATGAAATACATTTGTTCTGA	aaGAGCTCGGTACCTATCGATAGAGAAATGTTC
	pGL3－295/＋64	GCTAGGAGGGAAGTAGAATTTCAGTTACTTCAT	ttctacttccctcctagcGAGCTCGGTACCTATCGATAGAGAA
zip14	pGL3－1409/＋135	aggtaccgTTGTTAGTGGACTGTAATGTCACTGC	ccactaacaaCGGTACCTATCGATAGAGAAATGTTC
	pGL3－1153/＋135	ataggtaccgCTCTGTCAGTCCGTCTGTCTCTCC	tgacagaGCGGTACCTATCGATAGAGAAATGTTC
	pGL3－654/＋135	cgataggtaccgAAGACTTTATTACTTTGTTATAAGACACAAGTATG	agtcttCGGTACCTATCGATAGAGAAATGTTCT
	pGL3－266/＋135	gtaccgGCTGCGTTAAAAGACTAAAAAGTAATATT	ttttaacgcagcCGGTACCTATCGATAGAGAAATGTTC

5: The part of “uppercase” are primers to generate 5′ unindirectional deletion promoter sequence and pGL3-basic sequence using full-length promoters constructs as template, the part of “lowercase” are primers assistting to generate recombination sequence to recombinate the preduced deletion sequence and pGL3-basic sequence.

Table S3. Nested PCR primers used for the generating of pZip10-EGFP, pZip13-EGFP and pZip14-EGFP of *Pelteobagrus fulvidraco*

Genes	Group	Forward primer (5'-3')	Reverse primer (5'-3')
pZip10-EGFP	PCR-1	AAAGGGGTTTTCCGGTGTGTAGGAC	ACTCCGCTATAGCAGAAAGCAG
	PCR-2	[6]ctagcgtttaaacttaagcttATGGCAGGGACAAGCCGG	gctcaccatggtggcctcgagACTCCGCTATAGCAGAAAGCAGC
pZip13-EGFP	PCR-1	TACTGGCCTTAAAGCATTGGTCTCG	AGAAGTCGAAGTCGAACACAATGC
	PCR-2	ctagcgtttaaacttaagcttATGAGAGTTCACGTTCACACCAAG	gctcaccatggtggcctcgagAGAAGTCGAAGTCGAACACAATGC
pZip14-EGFP	PCR-1	TTCTCTGTGTTCTAGAGACATCGTT	ATCCGAGCTGTATCTGTCCTGAATA
	PCR-2	ctagcgtttaaacttaagcttATGTTACGGATAATGAGATCCTACAGA	gctcaccatggtggcctcgagATCCGAGCTGTATCTGTCCTGAA

6: The part of “uppercase” are primers to generate cDNA sequence coding the corresponding protein, the part of “lowercase” are primers assistting to generate recombination sequence to subclone to pcDNA3.1-EGFP plasmid.

Table S4. Primers used for site-mutation analysis

Gene	Primers	Forward primer (5'-3')	Reverse primer (5'-3')
zip10	MRE-*zip10*	[7]CTTtctgttcagaacggAAAAAAAAAAAACCTTTCACACCTT	TccgttctgaacagaAAGTCTGCTGTGAATTCAACCAGTT
zip13	MRE-*zip13*	AcacgtacagttcggcgAAACTTTGTAACGGAACGAAAGG	TcgccgaactgtacgtgTCCCTGAACCCCACATATTCA
	KLF4-*zip13*	tttgcatggagTTTGGCATGTAATAAGTGATTATTTATATTT	GCCAAActccatgcaaaTAATGCTTTATGTATCCATGGGCT
zip14	MRE-*zip14*	cgccgaactggatgtgTTGTACGTGTCATATGGTGTTATAAAGTG	AAcacatccagttcggcgGGGCTTTATTATCTATATTCATAATATTTATATATTT
	STAT(1)-*zip14*	agagagccggGATGTCATGTGTTTATTTCTGAAAAGAT	ATGACATCccggctctctGCAGTGACATTACAGTCCACTAACAA
	STAT(2)-*zip14*	GTTTAagaggctcgtcATTATTCTGTTATTCTCGTATGTTAGCAGC	TgacgagcctctTAAACACATGACATCTGACAGGAAAG

7: The part of "lowercase" are mutated unspecific binding sequence which was substituted according to Jaspar, the part of "uppercase" are sequence beside the predicted binding sequence.

Table S5. Primers used for electrophoretic mobility-shift assay (EMSA)

Primers		Forward primer (5'-3')	Reverse primer (5'-3')
MRE-*zip10*	Biotin-probe	Biotin-CTTGTGACTTGTGAAAAAAA	Biotin-TTTTTTTCACAAGTCACAAG
	Unspecific-competitor	CTTTCTGTTCAGAACGGAAA	TTTCCGTTCTGAACAGAAAG
MRE-*zip13*	Biotin-probe	Biotin-GGGAAATGAGTGTGCAACACAAAC	Biotin-GTTTGTGTTGCACACTCATTTCCC
	Unspecific-competitor	GCCCCACGTACAGTTCGGCGGTTT	AAACCGCCGAACTGTACGTGGGGC
MRE-*zip14*	Biotin-Probe	Biotin-GCCCTATTTGCACAGGGTTAGTTT	Biotin-AAACTAACCCTGTGCAAATAGGGC
	Unspecific-competitor	GCCCCGCCGAACTGGATGTGGTTT	AAACCACATCCAGTTCGGCGGGGC
KLF4-*zip13*	Biotin-probe	Biotin-GTACAGATGCACACTGTCATGC	Biotin-GCATGACAGTGTGCATCTGTAC
	Unspecific-competitor	GTATCACGATCAGTGAGTCTGC	GCAGACTCACTGATCGTGATAC
STAT3(1)-*zip14*	Biotin-probe	Biotin-ACTGCTTTCCTGTCAGATGT	Biotin-ACATCTGACAGGAAAGCAGT
	Unspecific-competitor	ACTGCAGAGAGCCGGGATGT	ACATCCCGGCTCTCTGCAGT

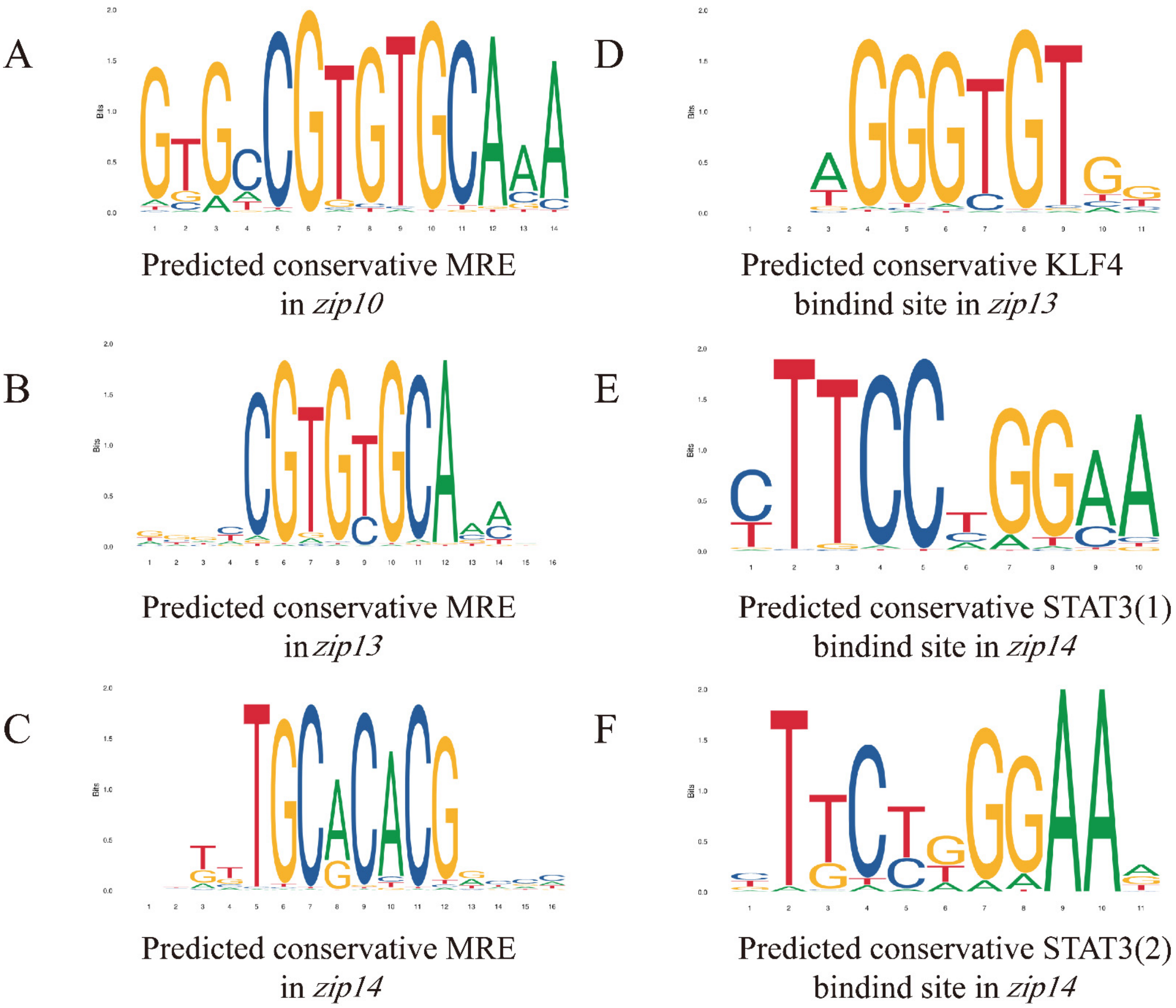

Figure S1. The sequences of the selected predicted TFBSs in *zip10*, *zip13* and *zip14* promoter of *P. fulvidraco*. The bigger a deoxynucleotide is, the more conservative a deoxynucleotide among vertebrates. Different color are only used to distinguish different deoxynucleotide. (A) Predicted conservative MRE of *zip10*; (B) predicted conservative MRE of *zip13*; (C) Predicted conservative MRE of *zip14*; (D) Predicted conservative KLF4 binding site of *zip13*; (E) predicted conservative STAT3(1) bind site of *zip14*; (F) Predicted conservative STAT3(2) bind site of *zip14*.
